# Diversity of RNA-Binding Proteins Modulating Post-Transcriptional Regulation of Protein Expression in the Maturing Mammalian Oocyte

**DOI:** 10.3390/cells9030662

**Published:** 2020-03-09

**Authors:** Marie Christou-Kent, Magali Dhellemmes, Emeline Lambert, Pierre F. Ray, Christophe Arnoult

**Affiliations:** 1 Université Grenoble Alpes, F-38000 Grenoble, France; marie.cck@gmail.com (M.C.-K.); magali.dhellemmes@univ-grenoble-alpes.fr (M.D.); emeline.lambert@univ-grenoble-alpes.fr (E.L.); PRay@chu-grenoble.fr (P.F.R.); 2 Institute for Advanced Biosciences INSERM U1209, CNRS UMR5309, F-38000 Grenoble, France; 3 CHU de Grenoble, UM GI-DPI, F-38000 Grenoble, France

**Keywords:** developmental competence, fertilisation, mRNA, oocyte maturation, translation

## Abstract

The oocyte faces a particular challenge in terms of gene regulation. When oocytes resume meiosis at the end of the growth phase and prior to ovulation, the condensed chromatin state prevents the transcription of genes as they are required. Transcription is effectively silenced from the late germinal vesicle (GV) stage until embryonic genome activation (EGA) following fertilisation. Therefore, during its growth, the oocyte must produce the mRNA transcripts needed to fulfil its protein requirements during the active period of meiotic completion, fertilisation, and the maternal-to zygote-transition (MZT). After meiotic resumption, gene expression control can be said to be transferred from the nucleus to the cytoplasm, from transcriptional regulation to translational regulation. Maternal RNA-binding proteins (RBPs) are the mediators of translational regulation and their role in oocyte maturation and early embryo development is vital. Understanding these mechanisms will provide invaluable insight into the oocyte’s requirements for developmental competence, with important implications for the diagnosis and treatment of certain types of infertility. Here, we give an overview of post-transcriptional regulation in the oocyte, emphasising the current knowledge of mammalian RBP mechanisms, and develop the roles of these mechanisms in the timely activation and elimination of maternal transcripts.

## 1. Introduction

For correct and timely function, each cell regulates the expression of its genes to suit its specific protein requirements at any given moment. A cell’s requirements differ according to cell type and depend on an interaction of extracellular and intrinsic cues. A cell can execute gene regulation at a number of stages along the protein production pathway: (1) At the transcription level, via transcription factors and chromatin accessibility, (2) at the mRNA level by controlling the processing, translation, and degradation of transcripts, and (3) at the protein level, where protein activity can be modulated via post-translational modifications and degradation.

The most prominent form of regulation for the majority of genes is executed at the level of transcriptional control [1]. The binding of transcription factors is influenced by epigenetic factors including DNA methylation, the histone composition of the nucleosome, and histone modifications. Transcription factors bind to accessible DNA regulatory sequences upstream of the gene near the site of transcription initiation through DNA-binding motifs and act as switches, turning on or off transcription through recruitment of the transcription machinery. Messenger transcripts then typically exist transiently, with eukaryotic transcript half-lives ranging from several minutes to several days [2]. In growing mouse fibroblast cells, the median average half-life was shown to be 9 h for mRNA compared to 46 h for proteins [3]. Protein expression depends on mRNA abundance, and mRNA abundance is determined by the balance of rates of transcription and decay. Factors that influence mRNA synthesis, stability, and association with the translation machinery thus have an important impact on protein expression.

Post-transcriptional regulation is an essential component of the cell’s ability to regulate protein expression as it represents a secondary layer of control that is independent from the transcription machinery. This type of regulation allows for rapid response to external stimuli [4] and becomes the prominent form of regulation in cases where transcription is not possible, i.e., due to compact/inaccessible chromatin. Post-transcriptional regulation can be global, through modulation of key components of the protein synthesis machinery, but it can also be RNA-specific, through the presence of *cis*-regulatory elements typically within the 3′UTR of the mRNA sequence. Such sequences are recognised by *trans*-acting RNA-binding proteins (RBPs), which are capable of recruiting or preventing recruitment of key actors of the synthesis machineries.

**Figure 1:**
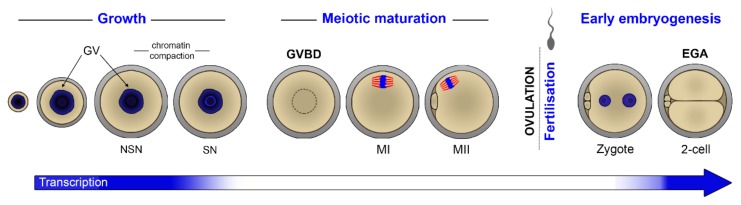
Oogenesis and early embryo development in mice. The blue colour in the arrow below indicates transcriptional activity. GV: Germinal vesicle, GVBD: Germinal vesicle breakdown, NSN: Non-surrounded nucleolus, SN: Surrounded nucleolus, MI: Metaphase I, MII: Metaphase II, EGA: Embryonic genome activation.

In the oocyte, a long growth phase during which chromatin is in an ‘open’ conformation and transcriptional activity is high is succeeded by a phase of resumed meiotic activity and transcriptional silencing. Meiotic resumption is triggered in fully grown GV oocytes in response to the LH surge, entailing chromatin condensation, completion of meiosis I (with extrusion of the first polar body), and progression through to metaphase of meiosis II before ovulation. At fertilisation, the oocyte completes the final stages of meiosis II (extruding the second polar body) whilst undergoing nuclear reprogramming and fusion of the male and female pronuclei as part of the maternal-to-zygote transition (MZT), followed by the first embryonic divisions and activation of the embryonic genome (EGA) (Figure 1). These numerous and complex processes essentially take place in the absence of new transcription. The unique challenge of the oocyte is therefore the synthesis and preservation of all transcripts necessary during this period of intense activity as well as their timely and selective translation and degradation.

To meet these requirements, a number of specialised RBPs exist, making up a specialised and multi-layered mRNA processing system. The oocyte environment is a particularly stable one, with a very low intrinsic decapping rate. Rather than hours, maternal oocyte mRNAs have an average half-life of around two weeks in growing mouse oocytes [5,6]. The preservation of maternal mRNAs and their regulated translation combined with the control of post-translational modification and protein degradation allow the oocyte to specifically modulate the abundance of active proteins in a temporal and spatial manner. mRNA regulatory networks can be found in oocytes across metazoan species. In this review, we give particular attention to the mechanisms that exist in mammalian (particularly mouse) oocytes.

## 2. The Journey of a Messenger RNA Guided by Bound Proteins

Throughout an mRNA’s lifetime, its movements and fate are governed by the proteins and other molecules that bind to it. An mRNA molecule is constantly bound by a multitude of RNA-binding proteins (RBPs) that are necessary for facilitating intracellular transport, entry to and exit from translational activity, and recruitment of or protection from the cell’s degradation machinery. For this reason, it is better to refer to the synthesis and movement of messenger ribonucleoprotein (RNP) particles. RBPs typically bind to elements within the mRNA sequence, often located in the 3′ UTR, through RNA-binding domains. Several such domains have been characterised, including zinc-finger (Znf), RNA recognition motif (RRM), and K homology (KH) domains. The affinity of these binding interactions is usually relatively low and the recognised sequence variable, leading to a range of possible affinities and interactions [7].

### 2.1. Pre-mRNA Synthesis and Processing

In eukaryotes, protein-coding genes with accessible promoter regions are transcribed by RNA polymerase II, which is recruited and bound to the promoter through transcription factors [8]. The polymerase synthetizes a pre-mRNA molecule from the DNA template in the 5′ to 3′ direction until beyond the gene’s stop codon. During elongation, the 5′ end of the RNA molecule receives a 7-methylguanosine cap, which prevents degradation by exonucleases and is bound by the nuclear cap-binding complex (CBC). In humans, the protein CPSF (cleavage and polyadenylation specificity factor) binds the AAUAAA sequence (also known as the polyadenylation signal or PAS) in the 3′ UTR as part of a complex that includes Poly(A) Polymerase (PAP). CPSF cleaves the transcript ~10-30 nucleotides after the PAS sequence, releasing the pre-mRNA and defining its end terminus [9]. PAP catalyses the addition of adenine bases to the 3′ terminus forming the 3′ poly-A tail of around 200 adenine nucleotides. This serves to protect the transcript from degradation and plays a major role in the regulation of translation initiation. Poly(A)-binding protein (PABP) binds to the poly(A) tail, protecting it from exonucleases. Non-coding introns are removed in a sequence-specific mechanism by the spliceosome. The mature messenger RNA and its associated proteins, forming a ribonucleoprotein (RNP) complex, can then be exported from the nucleus to the cytoplasm through recognition of the CBC by nuclear pore complexes [10].

### 2.2. Translation and Decay

**Figure 2:**
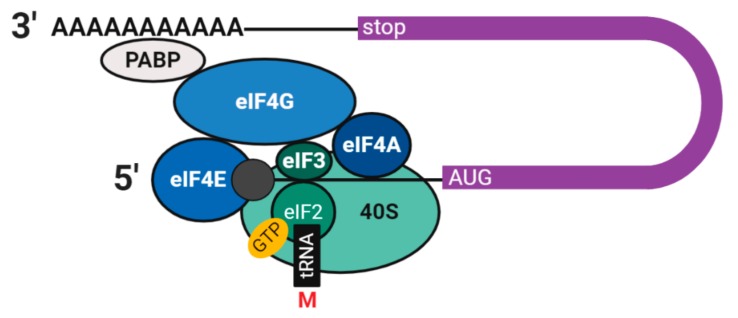
Translation initiation in eukaryotes. The closed-loop, cap-dependent translation initiation model. Proteins in shades of green form the 43S complex and in blue the eIF4F complex. eIF: Eukaryotic translation initiation factor, PABP: Poly(A) binding protein, M: Methionine, GTP: Guanosine-5′-triphosphate.

Once in the cytoplasm, the nuclear CBC is replaced by the trimeric cytoplasmic cap-binding complex, eIF4F, comprising eukaryotic translation initiation factors eIF4E (cap-binding), eIF4G, and eIF4A. eIF4F binds the 40S ribosomal subunit via eIF3. eIF4G associates with PABP, creating a pseudo-circular structure that favours efficient translation and protects the mRNA from degradation (Figure 2). Another initiation factor, eIF2 with bound GTP, recruits eukaryotic initiator tRNA (Met-tRNAi) to the 40S ribosomal subunit forming the pre-initiation complex. This complex locates and delivers Met-tRNAi to the AUG start codon, which with the aid of additional factors and GTP hydrolysis, recruits the 60S ribosomal subunit and assembles the full ribosome to begin translation [11,12]. mRNAs containing a premature stop codon are degraded with equal probability during each subsequent round of translation via nonsense-mediated decay [13].

A long poly(A) tail is correlated with translational efficiency. Deadenylation slows translation and, in most cell types, initiates mRNA degradation by disrupting the translation initiation complex and exposing the 5′ end to decapping enzymes. Deadenylation and exoribonuclease degradation is the pathway by which most mRNAs undergo decay. It is thought that first the PAN2/3 (poly(A)-nuclease) complex shortens the poly(A) tail to around 80 residues, after which the CCR4-NOT complex or PARN (Poly(A)-specific ribonuclease) takes over and removes most or all of the remaining tail [14]. Deadenylation is typically followed either by 3′ to 5′ degradation by the cytoplasmic exosome or association of the ring-shaped Lsm1–7 complex to the 3′ end, which induces decapping by Dcp1p/Dcp2p [15]. Decapping leaves the mRNA molecule vulnerable to 5′ to 3′ degradation by the exoribonuclease XRN1 [16]. mRNAs can also undergo decay via endonucleolytic degradation through sequence-specific cleavage by the RNA-induced silencing complex (RISC) associated with small interfering RNA (siRNA). The presence of certain AU-rich elements (AREs) in the 3′ UTR signals rapid degradation through specific binding proteins [17].

### 2.3. Somatic RNP Granules

**Figure 3:**
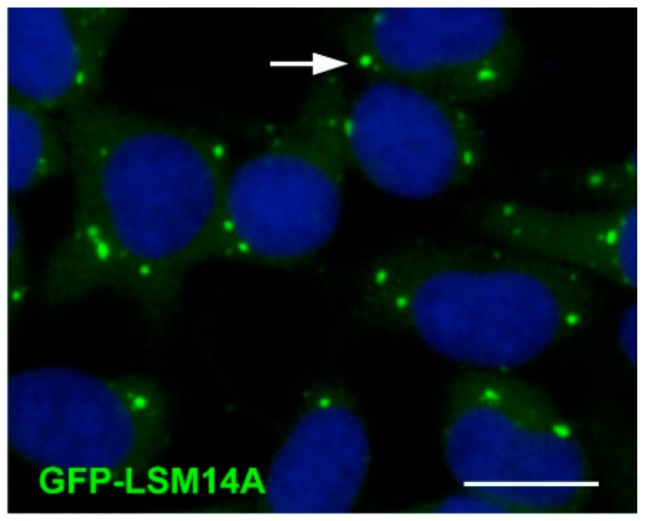
P-bodies labelled with GFP-LSM14A in HEK293 cells. The white arrow indicates a P-body. DAPI-stained nuclei are in blue. Scale bars: 10 μm. Source: Hubstenburger et al., 2017 [20].

The above-described mRNA decay and interference machinery as well as translationally inactive mRNAs tend to concentrate in discrete cytoplasmic foci known as P-bodies (processing bodies). P-bodies are membrane-less organelles that are sites of translational repression, mRNA degradation, and also mRNA storage, since mRNAs can subsequently return to translation [18]. They are phase-separated (liquid–liquid) RNP granules that present as droplets of around 0.5 µm (Figure 3). The role of P-bodies as sites of RNA decay or storage has been a subject of debate, however it is now thought that the primary function of P-bodies is the co-ordinated storage of regulatory mRNAs grouped by function, or ‘regulons’. The group of mRNAs that locates to P-bodies is particular in that they tend to have intrinsically low translation rates (lower polysome association and protein yield) [19]. P-bodies could then act to physically separate mRNAs requiring tight control and co-ordinated expression from those that are efficiently translated [20]. Mammalian proteins found to be indispensable structural P-body components include DDX6, 4E-T, and LSM14A/RAP55. Another type of somatic RNP granule are stress granules, which appear in response to environmental stress and contain mRNAs that are halted in translation initiation. They have a number of proteins in common with P-bodies such as CPEB1, DDX6, and eIF4E [21].

## 3. Oocyte RBPs and Post-Transcriptional Regulation

**Figure 4:**
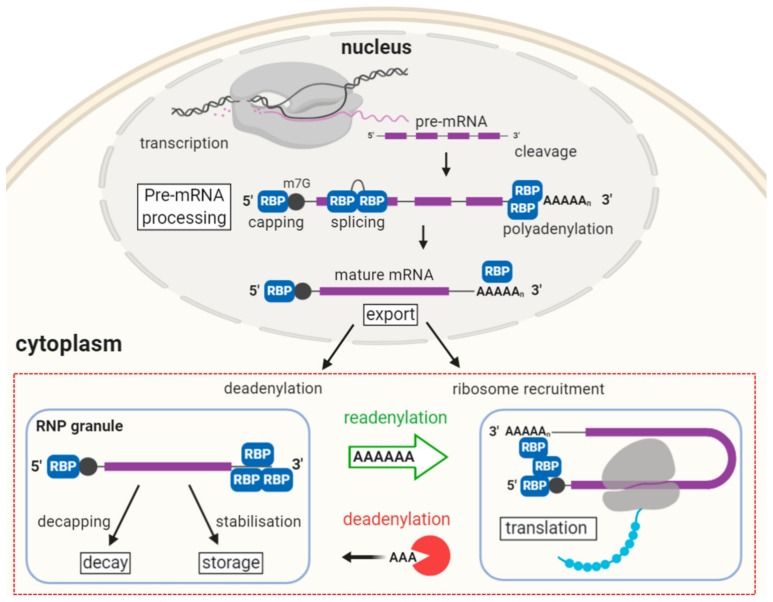
Nuclear and cytoplasmic post-transcriptional regulation in oocytes. The red box highlights the importance of cytoplasmic events in oocyte mRNA regulation. RBP: RNA-binding protein, RNP: Ribonucleoprotein. The purple block represents coding sequence and black circle represents the 7-methylguanylate (m7G) cap.

**Figure 5:**
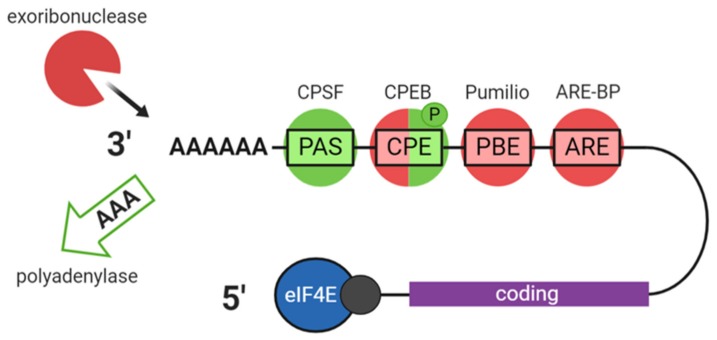
Key 3′UTR elements, their associated RBPs, and control of poly(A) tail length. 3′UTR elements and RBPs mediating polyadenylation and translation activation are shown in green and those mediating deadenylation and translational repression in red. PAS: Polyadenylation signal, CPSF: Cleavage and polyadenylation specificity factor, CPE: Cytoplasmic polyadenylation element, CPEB (CPEB1): CPE-binding protein, PBE: Pumilio-binding element, ARE: AU-rich element, ARE-BP: ARE-binding protein.

It is estimated that 30-45% of the mRNAs transcribed during oocyte growth in vertebrates are translationally repressed until meiotic maturation or fertilisation [22]. These stored mRNAs are typically deadenylated upon export from the nucleus to the cytoplasm, leaving poly(A) tails of around 20-40 residues long that are unconducive to translation since PABP and eIF4G are unable to bind [23]. In oocytes, deadenylation is not linked with decapping in the same way as in somatic cells [21]. The deadenylated state is preserved and the mRNA stabilised by the binding of specific RBPs until translation is required, at which time the mRNA is derepressed and the poly(A) tail is restored (Figure 4). This switch is often mediated by phosphorylation of the RBP as part of a signaling cascade. Some oocyte RBPs have a dual activity, both capable of repressing and activating/enhancing translation according to their phosphorylation state (Figure 5).

The most widely studied model organism for oocyte transcript dynamics is *Xenopus*, due to the quantity of oocyte material that can easily be obtained in comparison with mammalian models. This model has allowed the characterisation of many RNPs involved in translational control. RBPs whose mammalian orthologues have been shown to have important roles through mouse KO studies as well as any association to human reproductive disease are listed in Table 1. The most extensively characterised mechanism involves the cytoplasmic polyadenylation element (CPE) located in the 3′ UTR of many mRNAs and its binding protein CPEB. Another involves Pumilio (PUM) proteins that recognise the PUM-binding element (PBE) [24]. Figure 5 illustrates several key 3′UTR elements and their corresponding RBPs and whether they mediate translational repression or activation. Many other oocyte RNPs have been identified as playing important roles in oocyte mRNA regulation by mechanisms which have yet to be fully elucidated, such as Y-box-binding protein MSY2: A global regulator of mRNA stability and DAZL, which appears to be involved in translational activation by a polyadenylation-independent mechanism [6,25].

**Figure 6:**
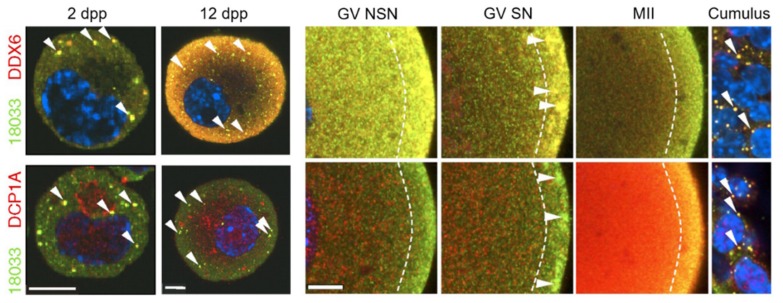
Germ-cell granules or ‘P-bodies’ in young mouse oocytes and a sub-cortical mRNA storage domain in growing/mature oocytes. Confocal images of mouse oocytes from 2 days postpartum (dpp) and 12 dpp females, immature NSN (non-surrounded nucleolus) and mature SN (surrounded nucleolus) GV oocytes, MII oocytes, and cumulus cells from adult females after staining with 18,033 (stains P-body protein EDC4), DCP1A, and DDX6 antibodies. Diagonal arrowheads depict P-bodies and horizontal arrowheads depict subcortical aggregates. Dashed lines border the subcortical domain. Staining with 18,033 is green, other proteins are red, and DNA staining in blue. Scale bars: 10 μm. Source: Flemr et al., 2010 [28].

RNP complexes represent spatial as well as temporal mRNA regulation, allowing for localised expression and degradation within the cell. The germ-cell equivalent of somatic P-bodies have been called ‘germ granules’, referring to cytoplasmic, membrane-less organelles that are unique to the germline and contain orthologues of somatic P-body proteins such as DDX6, suggesting a conserved role [26]. In many model organisms (*Xenopus*, *Drosophila*, *C. elegans,* and zebrafish), germ granules are present in oocytes of all stages. In mammals, however, germ granules have been described to exist only in primordial follicle oocytes of mice under two weeks of age [27,28]. One study by Flemr et al. [28] suggested that upon the disappearance of germ cell granules or P-bodies in mammalian oocytes, a new mRNA storage region appears close to the oocyte cortex. They showed that several RBPs (including DDX6, CPEB, and MSY2) transiently accumulate in RNA-containing subcortical aggregates in fully grown ‘SN’ or surrounded nucleolus GV oocytes (Figure 6).

Another type of RNA storage compartment identified in oocytes are Balbiani bodies: Large membrane-less cytoplasmic organelles that were initially described in dormant *Xenopus* oocytes in which they were found to contain Golgi elements, mitochondria, and endoplasmic reticulum as well as RNA [29]. Such structures have been reported to exist in oocytes of other vertebrate species, although in mice they have been identified only in very young oocytes, and these have not been found to contain RNA [30].

### 3.1. The CPEB1 Mechanism

CPEB1 functions as a crucial regulator of polyadenylation and therefore of translation. Originally characterised in *Xenopus* oocytes, it has seen been shown to regulate key mRNAs in various cell types including neurons [54]. It is a zinc finger-containing protein that assembles complexes with other proteins to perform a dual functionality, capable of both translational repression and activation according to its phosphorylation state. The CPE sequence recognised by CPEB1 is UUUUA(A)U. In a computational analysis of mRNA 3′ UTRs, 31% (mouse)–36% (human) of analysed mRNAs were predicted to be involved in CPE-mediated translational regulation [22]. CPEB1 is expressed until the MI stage in mouse oocytes and controls the translation of the oocyte-specific MAP kinase–kinase–kinase c-Mos and cyclin B1, proteins crucial for the progression of meiosis [25].

**Figure 7:**
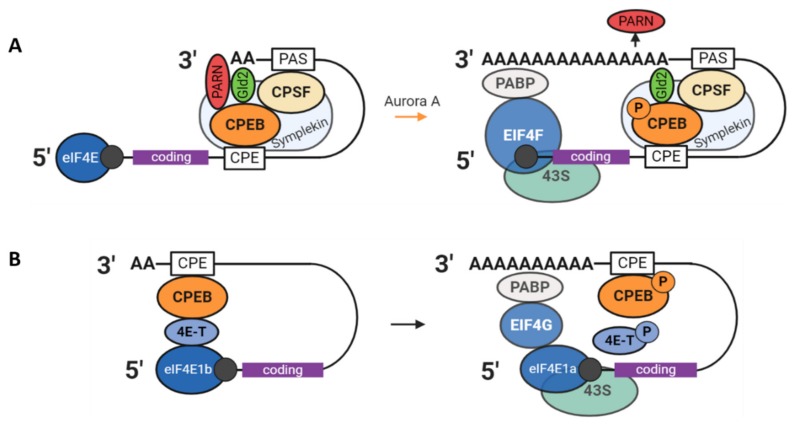
Models of regulation of mRNA translation by CPEB1 and associated proteins. (**A**) Poly(A) regulation via PARN and Gld2 as described in *Xenopus*. The transition from translational repression to activity occurs via phosphorylation of CPEB1 by the kinase Aurora A. Trimeric eIF4F comprises eIF4E, eIF4A, and eIF4G. (**B**) Proposed model of translation regulation through binding of 4E-T with CPEB1 and eIF4E1b hindering interaction of eIF4E1a with the cap structure.

In the CPEB1 mechanism, as characterised in frogs, poly(A) control is mediated through the following proteins: Symplekin (likely a platform protein), CPSF (bound to the 3′ UTR PAS sequence), poly(A) ribonuclease (PARN), and the poly(A) polymerase Gld2 (germ-line development factor 2). The latter two are both active with competing activities, however PARN has higher activity than Gld2, resulting in overall deadenylation of CPE-containing mRNAs. When Cpeb1 is phosphorylated by the Aurora A kinase (at Ser174), its binding to Gld2 and CPSF become stronger and, as a result, PARN is excluded from the RNP complex, allowing Gld2 to elongate the poly(A) tail (Figure 7A) [55]. The orthologous mechanism in mammals has yet to be fully characterised, especially in light of a study in 2007 that showed that GLD2 is dispensable in mouse oocytes [56]. Polyadenylation status was unaffected in the oocytes of *Gld2* KO mice as was overall health and fertility. This indicates evolutionary divergence and the existence of an alternative source of poly(A) adenylation in the mouse.

A second mode of CPEB1-mediated translational control characterised in *Xenopus* acts through the prevention of the interaction between cap-binding eIF4E and eIF4G via an intermediary protein, thus preventing formation of the eIF4F complex (Figure 7B). This intermediary protein was long thought to be Maskin [55] and the mechanism the following: when the poly(A) tail is short, Cpeb1 interacts with eIF4E via Maskin, then following Cpeb1 phosphorylation and mRNA polyadenylation, PABP binds to the poly(A) tail and binds eIF4G, which can outcompete Maskin and bind eIF4E allowing translation initiation. There is now, however, considerable evidence showing that the intermediary protein is more likely to be eIF4E transporter 4E-T, since Maskin is not expressed in growing oocytes and direct evidence of Maskin and eIF4E interaction is lacking [57,58].

4E-T (Clast4 in mice) is highly expressed in the cytoplasm of growing mouse oocytes and was identified as part of the Cpeb1 RNP in *Xenopus* oocytes alongside eIF4E [59]. It is phosphorylated during meiotic maturation in both *Xenopus* and mouse oocytes by an unknown kinase and is a partner of Xp54 (whose mammalian orthologue is DDX6). In *Xenopus* oocytes, 4E-T was shown to interact with eIF4E1b: An ovary-specific close homologue of eIF4E (70% identity) that weakly binds the 5′ cap [60] and whose expression is confined to oocytes and early embryos in *Xenopus*, zebrafish, and mice. A new model was proposed based on these observations for specific inhibition of translation via the eIF4E1b–4E-T–CPEB1 complex, which would seem to prevent the binding of eIF4E1a to the cap structure. In this model, which requires further validation, translation is inhibited through the repressive qualities of 4E-T (and associated Xp54) bound to the 3′UTR via unphosphorylated CPEB1, which is expelled upon CPEB1 phosphorylation allowing assembly of the eIF4F complex (Figure 7B) [21].

Another member of the CPEB family, CPEB4, has also been found to play an important role in oocyte meiotic progression. CPEB1 controls the translation of CPEB4, activating it at a later stage of meiosis. CPEB4 likely replaces CPEB1, driving the transition of metaphase I to metaphase II in a positive translational loop [33].

### 3.2. PUM2-DAZL-EPABP

The Pumilio proteins (PUM1/2), members of the PUF protein family, are involved in translational regulation in *Xenopus* oocytes. They bind to the 3′ PUM-binding element (PBE: UGUAX_2-4_UA) as well as the Nanos response element (NRE: UGUA) [7]. Pum1/2 are thought to mediate translational repression through interaction with the CCR4-NOT deadenylase complex [61]. Pum2 forms a complex with Dazl (Deleted in azoospermia-like) and Epabp (Embryonic poly(A) binding protein) and represses the translation of RINGO/Spy, a Cdk1 activator necessary for Cpeb1 and cyclin B1 activation in immature *Xenopus* oocytes [62,63]. PUM1 has recently been shown to be important in the establishment of the primordial follicle pool, meiosis, and oocyte developmental competence in mice [24]. There are reports that PUM1/2 can directly bind CPEB1 [62].

DAZL and EPABP can be considered co-repressors of translation when they are associated with PUM2. However, DAZL itself is as an activator of translation thought to be able to interact with a large number of oocyte RNAs through uracil-rich 3′ UTR sequences. In mice, DAZL is expressed until the zygote stage, plays an essential role in primordial germ cell differentiation, and is thought to regulate mRNAs important for spindle assembly, meiosis, and maternal mRNA degradation [34]. The proposed model for DAZL-mediated activation is via the recruitment of additional PABPs to the mRNA 3′ end, which facilitates additional interaction with the 5′ factors and enhanced recruitment of the ribosomal subunit [64].

While PABP acts as a global translational regulator, EPABP is specific to and is the predominant poly(A)-binding protein in oocytes and early embryos in *Xenopus*, mouse, and human [36]. Epabp prevents deadenylation and is required for translational activation of several key mRNAs in *Xenopus* oocytes. In *Xenopus,* Epabp is both part of the Cpeb1-CPSF and Dazl-Pum2 complexes. EPABP is required for oocyte maturation and also for oocyte-granulosa cell communication in mice [61,65].

### 3.3. PATL2

Pat1 (protein associated with topoisomerase II) proteins, conserved across eukaryotes, are RNA-binding proteins that have been shown to be involved in numerous aspects of mRNA metabolism. They participate in large regulatory RNP complexes and localise to P-bodies in somatic cells. The current hypothesis is that Pat1 proteins act as scaffold or platform proteins, interacting with both mRNA and other proteins of various functions. Pat1 proteins have been implicated in mRNA decay, translational repression, and, more recently, in pre-mRNA processing. A single Pat1 protein (Pat1p) exists in yeast and invertebrates, whereas in vertebrates, two orthologous proteins have evolved (PatL1/Pat1b and PATL2/Pat1a) with distinct regulatory functions in the soma and germline. Somatic PATL1 appears to be primarily involved in mRNA decay and PATL2, specific to oocytes, in translational repression.

*Xenopus* PATL2 (xPat1a) was identified as a cytoplasmic RNP in growing oocytes. It was found to be an abundant binding partner of Cpeb1, able to selectively bind RNA and to repress the translation of specific mRNAs. xPat1a is rapidly degraded at meiotic resumption and ectopic expression of xPat1a significantly impaired the ability of oocytes to mature upon progesterone stimulation [60,66,67]. In order to explore the role of mammalian PATL2, our laboratory has recently studied the effects of *Patl2* invalidation in mice. We showed that *Patl2* invalidation leads to subfertility in females with oocytes showing dysregulation of a number of key transcripts expressed at various stages of oocyte maturation [40]. Fewer oocytes from *Patl2* KO females mature to the MII stage and the rate of progression through the early embryo stages is significantly impacted. PATL2 therefore appears to be a crucial factor for oocyte maturation, although its precise mode of action and molecular interactions remain to be elucidated in mammals.

### 3.4. MSY2/FRGY2

Germ-cell specific RNA-binding protein MSY2 (Y-box binding protein 2, in *Xenopus* Frgy2) is highly abundant in mouse oocytes until fertilisation, comprising around 2% of total oocyte protein in mice [68]. Its *Xenopus* orthologue Frgy2 is a partner of Cpeb1 and a principal component of maternal RNPs [21]. In mice, it was shown that MSY2 depletion in GV oocytes leads to aberrant oocyte growth, reduced mRNA stability, failure to halt transcription, and a dramatic disturbance of the transcriptome. Thirty-six percent of oocyte transcripts were altered more than two-fold in *Msy2-/-* compared to WT oocytes, and there was a global decrease of 25% in mRNA quantity (despite failure to halt transcription) [6]. Since no consensus sequence was identified in the 3′UTRs of the dysregulated mRNAs, it was concluded that MSY2 likely acts as a ‘master regulator’ of global mRNA, conferring stability by an unknown, possibly non-sequence-specific mechanism.

### 3.5. DDX6 (Rck/p54)

DEAD-Box Helicase 6 is an RNA helicase with a diverse range of functions. It is both a decapping activator and translational repressor and is a component of all known types of RNP granules: P-bodies, stress granules, and germ cell granules [69]. In *Xenopus* oocytes, its orthologue Xp54 was shown to mask maternal mRNAs as a binding partner of Cpeb1, associating with mRNAs encoding proteins necessary for meiotic progression [70,71]. In transcriptionally active oocytes, Xp54 binds a major set of newly transcribed mRNAs in the nucleus and accompanies them to the cytoplasm by an alternative export pathway. In transcriptionally quiescent oocytes (beyond GVBD), Xp54 is restricted to the cytoplasm and remains associated with mRNAs until they are required for translation, either at meiotic maturation or after fertilisation. Its range of functions may be linked to a role as a remodeller of RNP complexes, as studies have suggested that it can displace RNA-binding proteins to accommodate others for entry into various pathways (degradation, storage, or decay). These functions are thought to be conserved in mammals, although functional studies are lacking [72].

### 3.6. Musashi (MSI)

Musashi RBPs have a double role as translational repressors and activators. They contain RNA recognition motifs in their N-terminals and bind to a Musashi binding element (MBE) in the mRNA 3′ UTR. Originally described in *Drosophila*, Musashi has been well-characterised in *Xenopus* oocytes where it was found to be part of the Pumilio and Cpeb1 pathway. RINGO/Spy-dependent Cdk1 activity phosphorylates Musashi, activating translation of its target mRNAs including Mos [73]. This activation requires association of Musashi with Epabp, and interaction between Musashi and the poly(A) polymerase Gld2 has been reported [73]. Musashi proteins have been shown to be expressed in mouse GV oocytes, however their role in mammalian oocytes remains poorly studied.

### 3.7. RAP55B/LSM14B

The RAP55 (mRNA-associated protein of 55 kDa, also known as LSM14) family consists of two paralogues in vertebrates: RAP55A and RAP55B. Conserved in eukaryotes, they contain several domains (including a LSm domain: A domain found in many RBPs conferring a specific 3D structure) allowing for translational repression, mRNP inclusion, and localisation to P-bodies and stress granules. Originally discovered as an RNA-binding in newt oocytes, xRAP55 has been found to repress translation of maternal mRNAs in *Xenopus* oocytes and interact directly with Xp54 [74]. It is expressed in *Xenopus* oocytes of all stages as well as in embryos, but was shown to be phosphorylated in matured oocytes, indicating a potential mode of action. In mouse oocytes, it has been identified in Balbiani bodies and found to colocalise with DCP1a and DDX6 [27,75]. Studies have primarily focused on RAP55A or have not made the distinction between the two paralogues, and their expression and functions have yet to be compared [76]. It was recently shown that whereas knockdown of RAP55A did not affect meiotic progression in mouse oocytes, RAP55B knockdown led to a high proportion of oocytes displaying metaphase I-arrest, chromosome misalignment, abnormal SAC (Spindle Assembly Checkpoint), and MPF (Maturation Promoting Factor) activation and modified Cyclin B1 and Cdc20 mRNA levels [37].

### 3.8. Somatic Cell-Mediated Translational Regulation

As is well established, the crosstalk between the oocyte and its surrounding cumulus cells is vital for the oocyte developmental competence. It is possible that this signaling affects translational regulation in oocytes. The cumulus-cell-dependent PI3K/PTEN/mTOR signaling pathway influences the accumulation of certain oocyte proteins. During GVBD, mTOR kinase (TORC1) downstream of cumulus cell growth factor/Kit Ligand stimulation phosphorylates 4E-BP: A cap-dependent translational repressor expressed in mammalian oocytes. This leads to 4E-BP’s detachment from eIF4E allowing for translational activation. Transcripts that are controlled via this pathway include those required for spindle assembly, chromosome alignment, and segregation, such as TPX2 (Targeting Protein for the *Xenopus* kinesin xklp2) whose translation is controlled in a spatial as well as temporal manner [77,78]. The subset of mRNAs controlled in this way may be determined by the presence of TOP (terminal oligopyrimidine) sequences in the 5′ UTR of transcripts [77]. Differentially phosphorylated forms of 4E-BP have been identified on the meiotic spindle and at the spindle poles in mouse oocytes, and its phosphorylating kinases are also present on the spindle at the beginning of meiotic resumption, indicating that phosphorylation of 4E-BP promotes translation of spindle assembly components. Expression of a mutant form of 4E-BP results in spindle abnormality [79].

### 3.9. Small Non-Coding RNAs

Small non-coding RNAs such as microRNAs (miRNAs) and endogenous small interfering RNAs (endo-siRNAs) are important actors of post-transcriptional control and are expressed in mammalian oocytes. These small RNAs degrade or repress the expression of target mRNAs through full or partial sequence complementarity. miRNAs are transcribed by RNA polymerase III in a precursor form and processed by the Drosha/DGCR8 complex before being exported to the cytoplasm and cleaved by DICER to around 21 nucleotides. They then combine with Argonaute (AGO) proteins to form an RNA-induced silencing complex (RISC) that recognises the 3′ UTR of targets. siRNAs differ to miRNAs in that they bypass Drosha/DGCR8 processing, require full sequence complementarity, and associate with AGO2, which is unique in having endonucleolytic activity.

miRNAs have been found to play a critical role in deadenylation and clearance of maternal mRNAs in zebrafish zygotes [80]. In mice however, whereas invalidation of *Dicer* or *Ago* leads to disrupted meiotic maturation and spindle formation defects [61], *Dgcr8* invalidation (interfering with miRNA but not siRNA levels) did not affect oocyte or embryo development [81]. This indicates potential species differences and a particularly important role for endo-siRNA regulation in mouse oocytes, also known to be highly active [82]. Based on these contradictory results, the role of miRNAs during oogenesis remains to be clarified.

## 4. Waves of Translation and Degradation

### 4.1. Translational Activation after GVBD

Oocyte maturation is governed by waves of translation leading to the cascade of molecular events that are associated with meiotic resumption. CPEB1-mediated activation and polyadenylation of dormant mRNAs is thought to play a primary role. Many elements of this cascade have been elucidated in *Xenopus* oocytes, in which meiotic progression can be induced through treatment with progesterone. The chain of events leading to translational activation is not as well characterised in mammals as in amphibians due to difficulty in obtaining sufficient material. Although largely similar, certain differences, especially in the timing of events, have been reported between the amphibian and mammalian mechanisms, including later phosphorylation of CPEB1 in mammals.

Meiotic progression requires activation of kinases MAPK (mitogen-activated protein kinase) and CDK1 (cyclin-dependent kinase 1). The MAPK cascade is activated by Mos, which also combines with CDK1 to form the Maturation Promoting factor. Translation of both Mos and CDK1 is activated by CPEB1 [55]. CPEB1 activation, which occurs first near the oocyte membrane, is mediated by Aurora A kinase. The upstream events of Aurora A kinase-mediated CPEB1 activation depend on the key protein RINGO/Spy (Rapid inducer of G2/M progression in oocytes/Speedy) and its regulation via the polyadenylation-independent Pumilio-EPABP-DAZL mechanism. In *Xenopus*, this mechanism is as follows: upon progesterone stimulation, Pum2 (Pumilio) dissociates from the Pumilio Binding Element present in the 3′ UTR of RINGO/Spy mRNA as well as from Dazl and Epabp. The Dazl–Epabp complex then activates RINGO/Spy translation and RINGO/Spy recruits Cdk1, initiating the phosphorylation of Aurora A as well as Musashi. Aurora A kinase phosphorylates Cpeb1, and Musashi induces early activation of Mos and cyclin B5 [63,73,83]. Phosphorylation of Cpeb1 leads to the polyadenylation and translation of CPE-containing mRNAs by the mechanisms previously described.

**Figure 8:**
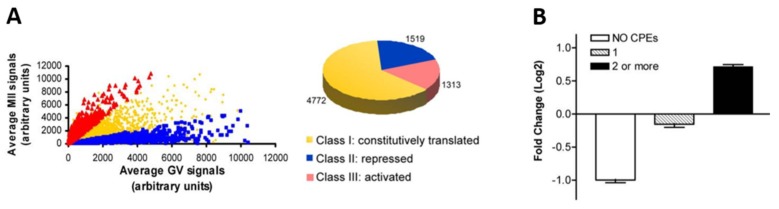
The oocyte translation program. (**A**) Comparison of transcripts associated with the polysome in GV and MII mouse oocytes divided into three classes: Constitutively translated (yellow), repressed (blue) or activated (red) between the two stages according to a two-fold change cut-off. (**B**) The relation between the number of CPEs in an analysis of the 3′ UTRs of 4645 transcripts and log2-fold change in polysome association between GV and MII. Source: Chen et al., 2011 [34].

One mouse oocyte study analysed polysome-bound mRNA and revealed a switch in the translation program of mouse oocytes before and after GVBD (in MII versus GV oocytes) [34]. One-third of all analysed mRNAs were recruited to the polysome (upregulated or translationally activated) or exited the polysome (downregulated or translationally repressed) by at least a two-fold factor upon meiotic maturation (Figure 8A). The 3′ UTR sequences of the translationally activated mRNAs were found to contain two or more CPEs, whereas those lacking CPEs were associated with downregulation (Figure 8B). The same pattern was found for DAZL-binding elements. The authors carried out a functional annotation of the genes whose polysome association was significantly altered between GV and MII stages. Amongst the list of genes exiting the polysome between these stages were genes involved in metabolism, DNA repair, protein biosynthesis, and transport as well as ribonucleoprotein genes. Transcripts recruited to the polysome included components of the cell cycle and transcription machinery and genes involved in chromatin remodeling and mRNA processing/decay [84].

Differences have been found in the timing of polyadenylation of CPE-containing mRNAs during maturation such as for Mos and Cyclin B1. Such differences could be explained by a combinatorial code of 3′UTR *cis*-elements and their *trans*-binding proteins whereby the state and timing of translational repression or activation is determined by a play-off between repressive and activating factors. Piqué et al. [22] described a code based on the number and relative position of CPEs and PBEs that determined the timing of Cpeb1-mediated translation in frog oocytes. A more recent study defined such a code in mouse oocyte mRNAs whereby the efficiency of translational repression and activation depended on the numbers and positions of CPEs relative to PASs [85]. Differentially timed translation activation can also be mediated by the sequential activation of RBPs, such as has been proposed for DAZL which is thought to be translationally activated during oocyte maturation by CPEB1 [34]. In this model, DAZL could be responsible for a second wave of translational activation of transcripts necessary during later stages of meiosis, in a chain that grants another layer of temporal control.

### 4.2. Degradation of Maternal Elements

**Figure 9:**
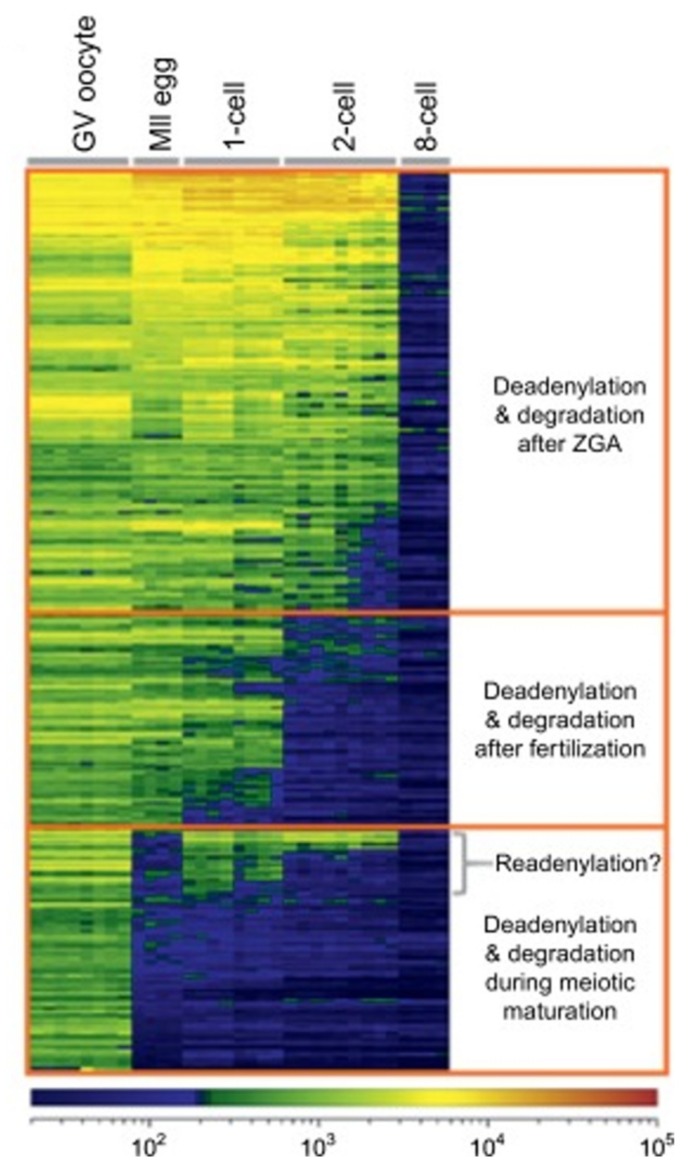
Microarray profiling of ~300 oocyte-specific genes during maturation. The color scale indicates mRNA abundance according to fluorescence intensity (arbitrary units). ZGA: Zygotic genome activation. Source: Svoboda et al., 2015 [5].

The degradation of maternal mRNAs begins at meiotic resumption. In mouse oocytes, meiotic resumption is accompanied by a decrease in total poly(A) RNA quantity from 80 pg to around 50 pg per oocyte. This degradation continues in the zygote and two-cell embryo, where it drops to 10 pg [6]. As shown Figure 9, meiotic resumption, fertilisation, and EGA each trigger waves of mRNA degradation. The progressive purging of maternal mRNAs is a requirement in order to allow the embryonic genome to take control of gene expression at EGA. Disruption of the waves of mRNA degradation obstruct embryonic development [7].

Degradation of maternal transcripts is highly regulated both temporally and spatially according to a species-specific program [86]. Generally, both maternal factors and newly transcribed zygotic factors (from the minor EGA) are involved in this process [87]. In *Xenopus*, the maternal component of the decay machinery mainly involves RNA-binding proteins that recognise 3′ UTR sequences such as AREs (AU-rich element) and EDENs (embryonic deadenylation element). The EDEN binding protein (EDEN-BP) is activated and induces deadenylation upon fertilisation. Degradation of targeted maternal mRNAs then depends on the zygotic component that largely relies on small RNAs [86]. A well-characterised example in *Drosophila* is the maternal RBP Smaug, which deadenylates maternal mRNAs through recruitment of the CCR4-NOT complex [88].

In mice, there is a strong wave of transcript elimination that occurs shortly after fertilisation triggered by maternal factors, and a second round that coincides with the major EGA mediated by zygotic factors [89]. The program that determines specific maternal transcript degradation in mammals is, however, poorly defined. One mechanism that is well characterised in mammals is the global transition to instability via MSY2 phosphorylation. MSY2, which confers stability to mRNAs during oocyte growth, is phosphorylated upon meiotic maturation by a CDK1-mediated means, such that virtually all MSY2 is phosphorylated by the MII stage [90]. The precise effect of this phosphorylation on the function of MSY2 is not clear, however the consequence is the degradation of maternal mRNAs, presumably due to loss of stability. It is likely that the phosphorylated form of MSY2 is unable to bind or otherwise protect mRNAs from the oocyte’s degradation machinery [91]. MSY2 mRNA levels also drop leading to a decrease in MSY2 protein, which remains detectable at low levels until the two-cell embryo stage.

Maternal proteins are also targeted for degradation during the maternal-to-zygote transition (MZT) by the ubiquitin–proteasome pathway [92]. The removal of germline-specific proteins and proteins involved in the MZT is an important step as many of these would interfere with the acquisition of a somatic, totipotent identity and mitotic divisions if they remained. This is another step in the erasing of the maternal legacy and handing over of control to the embryonic program.

## 5. RBPs and Human Infertility

As shown in Table 1, several of the RBPs discussed here have been associated with infertility in humans, with the majority being associated with cases of primary ovarian insufficiency (POI). POI refers to a loss of ovarian function before the age of 40. In the large majority of cases, POI is associated with premature depletion of the reserve of primordial follicles by an unknown mechanism due to accelerated follicular recruitment, activation of proapoptotic pathways, or autoimmune response [93]. However, in rare cases, the ovarian reserve is preserved, and problems arise in the later stages of follicular recruitment, maturation, and ovulation. Genetic causes are identified in around 20%-25% of cases [32].

In a cohort study of 259 POI patients, 1.3% of patients were found to harbour a heterozygous chromosomal microdeletion affecting the *CPEB1* gene, indicating CPEB-haploinsufficiency as a rare cause of POI [32]. Another study identified both heterozygous and homozygous single nucleotide polymorphisms (SNPs) of *DAZL* in cohorts of women with POI and men with azoospermia (few or no sperm in the ejaculate). *PUM2* SNPs have been associated with POI in a genome-wide association study (GWAS) [48]. Variants of *EIF2B* genes, part of the 43S translation preinitiation complex (Figure 2) (not listed in Table 1 since mRNA-binding is indirect) has also been associated with POI [94].

A rarer type of human infertility is Oocyte Maturation Deficiency (OMD), a type of primary infertility characterised by the repetitive production of immature oocytes that do not mature in vitro. Invalidation of *PATL2* has recently been associated with OMD in cases across several continents [40,41,42,43,44], with biallelic mutations associated with a GV-arrest phenotype and compound heterozygous mutations to a range of phenotypes from GV to early embryo arrest.

Several other oocyte RBPs with important roles in human oogenesis exist, for example *FMRP* and *LIN28*, whose dysregulation have been associated with POI and germ cell tumours respectively [95]. These have not been discussed in this review since their functions are during the stages of germ cell differentiation and follicle formation rather than during oocyte maturation.

## 6. Conclusions and Perspectives

It is clear that RBPs play an indispensable role in oocyte maturation and developmental competence across species. Given their importance, these complex regulatory networks merit further clarification, particularly in mammalian systems for which knowledge is often lacking. There are many aspects of oocyte translational regulation that require further elaboration, for instance, the spatial aspect of mRNA regulation, the exact role of small non-coding RNAs and the mammalian networks orthologous to those described in amphibians or invertebrates. The species differences that have been found between orthologous mRNA regulatory systems show that, if we want to improve our knowledge of human oogenesis, it is important to identify models whose mechanisms most closely resemble the human system despite the challenges involved.

The existence of oocyte-specific RBPs and their importance for oocyte maturation would make them good candidates for association with female infertility in cases of gene invalidation or with oocyte ageing. Infertility is a global public health issue affecting over 48.5 million couples worldwide. The elucidation of these RNA regulatory networks generates knowledge that could therefore be used to improve our diagnostic capabilities for human infertility and may aid in the development of new treatment strategies. Beyond this, the knowledge generated can be used to inform the improvement of existing assisted reproductive techniques such as in vitro oocyte maturation (IVM).

IVM is a technique currently applied to immature retrieved oocytes for IVF (in vitro fertilisation) or ICSI (intracytoplasmic sperm injection) in which oocytes are cultured to induce oocyte maturation to make fertilisation possible. The success of this technique is intimately dependent on culture conditions, and its effectiveness is currently limited to oocytes derived from larger follicles. A better understanding of the processes that occur during oocyte growth and maturation could inform the optimisation of culture conditions, improving success rates and potentially paving the way for expansion of this technique to the maturation of younger, more immature oocytes. This would have far-reaching applications, including creating the possibility for fertility preservation for young cancer patients whose oocytes may be cryopreserved ahead of reprotoxic therapy.

In this review, we have highlighted the importance of oocyte RNA-binding proteins in post-transcriptional regulation during and beyond oocyte maturation. The correct accumulation of maternal RNAs and RBPs during the growth phase and the finely balanced control that recruits RNAs to the translation apparatus or initiates their degradation in a timely fashion are processes that are central to the production of developmentally competent oocytes. We must strive to understand the oocyte’s complex regulatory mechanisms if we are to understand the pathologies linked to their disruption, and to recreate conditions favorable to correct oocyte development in reproductive therapy.

## Figures and Tables

**Table 1:** Mammalian oocyte RNA-binding proteins (expressed in maturing oocytes) with corresponding transgenic mouse phenotypes and any association of gene variants with human reproductive disease (female). POI: Primary ovarian insufficiency, OMD: Oocyte Maturation Deficiency, GWAS: Genome-wide association study.

Gene Name	Mouse Phenotypes	Association to Reproductive Disease in Women
*CPEB1* Cytoplasmic polyadenylation element binding protein 1	KO: females sterile. Embryonic oocyte development suspended at the pachytene stage of prophase I [31]	Heterozygous deletion associated with POI [32]
*CPEB4* Cytoplasmic polyadenylation element binding protein 4	KD: oocytes show impaired MI to MII transition and absence of first polar body extrusion [33]	-
*DAZL* Deleted in azoospermia-like	KD: oocytes showed decreased translation during late meiosis and improper spindle assembly KO: females (and males) sterile [34]	Missense mutations associated with POI in homozygous and heterozygous states [35]
*EPABP* Embryonic poly(A)-binding protein	KO: females infertile. Impaired growth and lack of transcriptional silencing in GV oocytes; failed translation activation in MII oocytes [36]	-
*LSM14B* LSM Family Member 14B	KD: high proportion of metaphase-1 arrested oocytes [37]	-
*MSI2* Musashi homolog 2 (*Drosophila*)	KO: females showed impaired folliculogenesis and decrease in number of MII oocytes [38]	-
*MSY2* Y-box-binding protein 2	KO: females (and males) infertile with impaired folliculogenesis and oocyte loss [39]	-
*PATL2* Protein associated with Topoisomerase II (yeast)-like 2	KO: females subfertile, high incidence of 1) oocytes not maturing to MII stage, 2) aberrant response to fertilisation and 3) developmental arrest before blastocyst stage [40]	Nonsense, missense, frameshift and splicing variants associated with OMD with GV arrest for homozygous variants and GV to early embryo arrest for compound heterozygous mutations [40,41,42,43,44]
*PUM1* Pumilio 1 (*Drosophila*)	KO: diminished ovarian reserve, oocytes showed delayed meiosis [24], higher rate of abnormal embryo development [45]	No pathogenic variants identified in study of POI patients [46]
*PUM2* Pumilio 1 (*Drosophila*)	KO: Females of normal fertility, possible redundancy with PUM1/species difference [47]	GWAS association with POI [48]
*ZAR1/2* Zygote arrest 1/2	ZAR1 KO: failed cleavage after fertilisation, some blastocysts with impaired EGA [49] ZAR1/2 KO: delayed meiotic resumption and first polar body extrusion, high frequency of abnormal meiotic spindle and chromosome aneuploidy [50].	-
*ZAR1L* Zar1-like	KD: two-cell stage embryonic arrest [51]	-
*ZFP36L2* mRNA decay activator protein	KO (C57BL/6NTac): embryonic arrest at the two-cell stage KO (F3 strain): females anovulatory, oocytes did not mature [52,53]	-
